# Retraction Note: ERK1/2-Nanog signaling pathway enhances CD44(+) cancer stem-like cell phenotypes and epithelial-to-mesenchymal transition in head and neck squamous cell carcinomas

**DOI:** 10.1038/s41419-024-06608-7

**Published:** 2024-03-15

**Authors:** Chuang Huang, Changhwan Yoon, Xiao-Hong Zhou, Ying-Chun Zhou, Wen-Wen Zhou, Hong Liu, Xin Yang, Jun Lu, Sei Young Lee, Kun Huang

**Affiliations:** 1 Chongqing Key Laboratory of Translational Research for Cancer Metastasis and Individualized Treatment, Chongqing University Cancer Hospital & Chongqing Cancer Institute & Chongqing Cancer Hospital, Chongqing, China; 2 Gastric and Mixed Tumor Service, Department of Surgery, Memorial Sloan Kettering Cancer Center, New York, NY USA; 3 Department of Gastric Surgery, Fujian Medical University Union Hospital, Fujian Province Chongqing, China; 4 Department of Otorhinolaryngology-Head and Neck Surgery, College of Medicine, Chung-Ang University, Seoul, Korea; 5 Department of Dermatology, The First Affiliated Hospital of Chongqing Medical University, Chongqing, China

Retraction to: *Cell Death and Disease* 10.1038/s41419-020-2448-6, published online 23 April 2020

The Editors-in-Chief have retracted this article after concerns were raised about image irregularities. Further investigation by the publisher found the below concerns to be valid. Additionally, the authors failed to provide raw data and evidence for ethics approval for this study.

The Editors-in-Chief therefore no longer have confidence in the results and conclusions present in this article.

- Partial image overlap between Figure 2A (sh. NANOG, SCC-15 CD44+ cells) and Figure 4G, Migration (SCC-25 CD44+ cells, sh.ERK1/2)
- Image overlap between Figure 2E (sh.SCr, SCC-15 CD44+ cells) and Figure 3C (SK-LMS-1, Spheroids) of [1]
- Image overlap between Figure 2E (sh.SCr, SCC-25 CD44+ cells) and Figure 3C (HT1 080, Spheroids) of [1]
- Multiple image overlaps between Figure 4H, immunofluorescent images and Figure 1D (SNU-668, Spheroids +) of [2]

Kun Huang agrees to retraction. None of the remaining authors have responded to any correspondence from the publisher about this retraction.
